# The effects of laparoscopic graspers with enhanced haptic feedback on applied forces: a randomized comparison with conventional graspers

**DOI:** 10.1007/s00464-017-5623-9

**Published:** 2017-06-07

**Authors:** Chantal C. J. Alleblas, Michel P. H. Vleugels, Sjors F. P. J. Coppus, Theodoor E. Nieboer

**Affiliations:** 1 Department of Obstetrics and Gynecology (791), Radboud University Medical Center, P.O. Box 9101, 6500 HB Nijmegen, The Netherlands; 2 Department of Obstetrics and Gynaecology, Riverland Hospital, Tiel, The Netherlands

**Keywords:** Technology, Laparoscopy, Innovation, Usability, Haptic feedback, Experimental research

## Abstract

**Background:**

Haptic feedback, which enables surgeons to perceive information on interaction forces between instrument and tissue, is deficient in laparoscopic surgery. This information, however, is essential for accurate tissue manipulation and recognition of tissue consistencies. To this end, a laparoscopic grasper with enhanced haptic feedback has been developed: the force reflecting operation instrument (FROI). This study tested the effects of enhanced haptic feedback on force control, tissue consistency interpretation, and the associated surgeons’ level of confidence through a randomized controlled crossover experiment.

**Methods:**

A randomized three-period crossover trial was conducted, in which seven surgical residents and 13 medical students participated. The setup involved a box trainer in which slices of porcine organs (lung, small intestine, or liver) were presented. Participants performed three series of blinded palpation tasks involving three different graspers: the conventional grasper, the FROI with enhanced haptic feedback activated, and the FROI with enhanced haptic feedback deactivated. In each series, nine pairs of organ tissues were palpated to compare consistencies. The orders of presenting both instruments and tissues were randomized.

**Results:**

The force applied during tissue palpation significantly decreased, by a mean factor of 3.1 with enhanced haptic feedback. Tissue consistency interpretation was significantly improved with more correct assessments and participants answered with significantly more confidence when enhanced haptic feedback was available.

**Conclusion:**

The availability of enhanced haptic feedback enabled participants to operate with significantly reduced interaction force between instrument and tissues. This observation is expected to have multiple important clinical implications, such as less tissue damage, fewer complications, shorter operation times, and improved ergonomics.

Since the early 1990s, when the implementation of laparoscopic surgery began to increase, its complexity has been highlighted [1, 2]. In addition to the reduced degrees of freedom in instrument movement, interference from cameras and other instruments has eliminated direct visual feedback and haptic feedback. However, technological advances [3] and sophisticated equipment have found their way into clinical practice, evident from improvements in visual feedback [4] and the introduction of robotic surgery [5]. Nevertheless, haptic feedback is still deficient in conventional laparoscopy, and it is completely lost in robotic surgery. The implementation of haptic feedback in laparoscopic instruments has not yet found its way into clinical practice. This is remarkable because explicit attention was drawn to this topic over a decade ago [6–8] and the hand-assisted laparoscopic surgical technique was introduced in the late 1990s especially for the benefits of direct tissue palpation [9–11]. Furthermore, several technological efforts have focused on the problem [12].

Introducing enhanced haptic feedback might well be the next big advancement in laparoscopic surgery, and both patients and surgeons stand to benefit [13, 14]. From a broad perspective, haptics involves the sense of touch and human interactions with the environment through touch. Haptic perception incorporates tactile and kinaesthetic perception. Tactile perception is based on receptors in our skin, which detect pressure, vibration, and texture. Kinaesthetic perception is based on receptors in our muscles, tendons, and joints. They detect position, movement, and force [15, 16]. When translating this to surgery, haptic perception is essential for accurate tissue identification and for accurate control over applied forces during tissue manipulations. These two abilities have been specifically acknowledged as important by laparoscopic specialists [17]. Up to now, in laparoscopic surgery, the surgeon has to rely on visual feedback and experience to estimate the appropriate amount of grasping force. Moreover, it has been reported that visual cues can be interpreted, with experience, as haptic information [18]. However, it was also found that providing both visual and haptic feedback could lead to better performance than either visual or haptic feedback alone [19].

Previous experimental studies have revealed that haptic feedback was significantly reduced in laparoscopic surgery compared to open surgery. Ottermo et al. found that the use of laparoscopic graspers decreased the accuracy of tissue recognition by fivefold [20]. Den Boer et al. reported that the perception of pulsation was reduced by at least a factor of 8 [21]. Heijnsdijk et al. found that the applied grip force in laparoscopy was at least twofold higher than necessary to manipulate tissue [22]. Those results suggested that, although it may be possible to receive some haptic feedback from laparoscopic graspers, the amount of haptic feedback about tissue properties and tissue reactions lacks clinical relevance for delicate tissue manipulation. With this study, we aimed to investigate the effects of enhanced haptic feedback on force control, tissue consistency interpretation, and associated surgeons’ level of confidence.

## Materials and methods

A special technique, involving “optical fiber Bragg grating” technology, has been deployed to introduce haptic feedback in a laparoscopic grasper that can be used in a clinical setting [23]. A prototype laparoscopic grasper with enhanced haptic feedback has been developed, called the force reflecting operation instrument (FROI). This instrument is capable of measuring the force applied on tissue with the instrument tip and transmits this information to the surgeon through a resistance mechanism in the instrument handle.

### Participants

Residents with laparoscopic experience were recruited through a request directly distributed by e-mail to all gynaecological residents affiliated with the Radboud University Medical Center, Nijmegen, The Netherlands. Additionally, medical students were recruited through a similar request placed on the medical faculty’s online bulletin board. Both requests stated the aim of the study and provided a summary of the experimental study design.

### Experimental design and procedure

Experiments took place in the Central Animal Laboratory (CDL), Nijmegen, The Netherlands. This study was designed in consultation with an animal welfare officer and a zoological technical analyst affiliated with the CDL. No approval from the Dutch Central Committee on Animal Studies (CCD) was required, because no live animals were used in this study. Fresh porcine organ tissue (slaughterhouse material) was provided by the CDL and processed according to the CDL regulations.

**Fig. 1:**
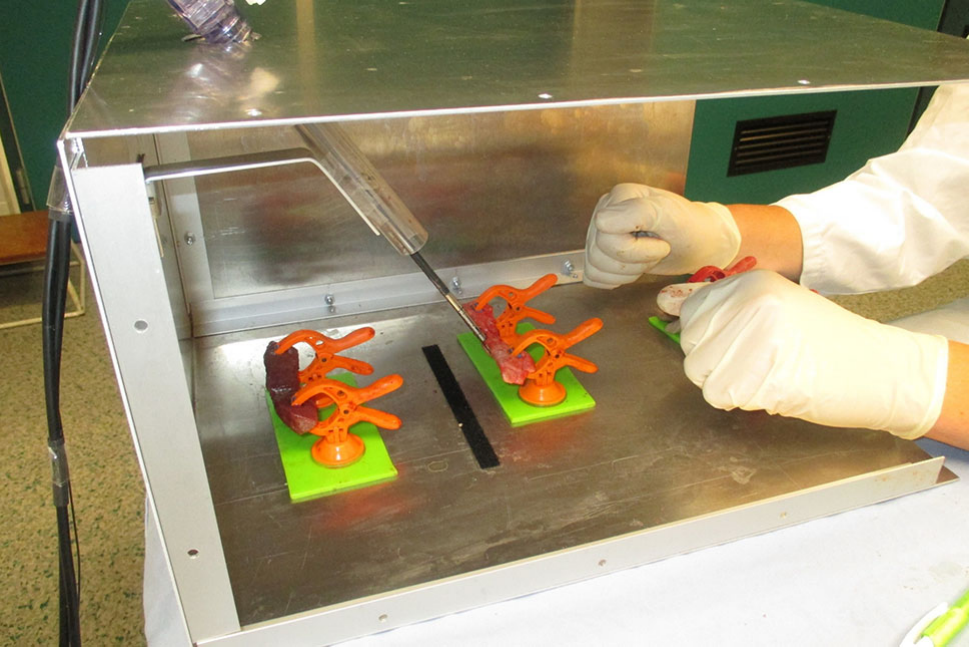
Experimental setup of the box trainer. The participant stands on the *left* for holding the grasper, and the instructor on the *right* for placing the tissues in front of the grasper tip

**Fig. 2:**
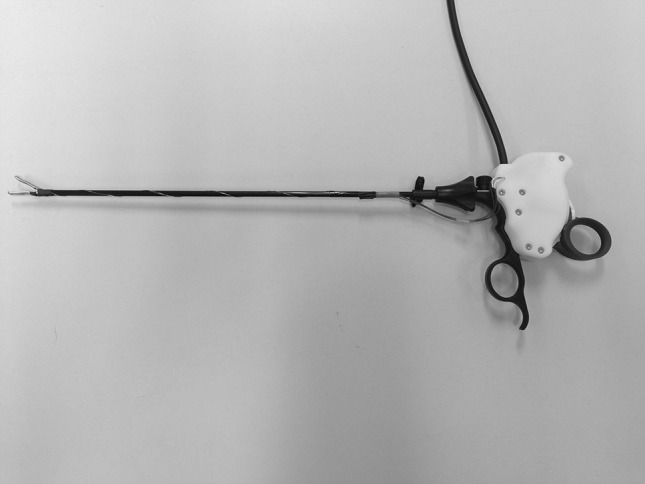
The force reflecting operation instrument (handle type: back hinged scissors)

The experimental setup involved a box trainer. Fresh slices of porcine organs (lung, small intestine, and liver) were presented in the box (Fig. 1). Before the trial was conducted, the appropriateness of these tissues was assessed by two laparoscopic experts and two novices. All four were able to distinct lung from small intestine and liver and vice versa while palpating the tissues with a gloved hand (as in open surgery) and without any visual feedback. Furthermore, the FROI technology allows the surgeon to predefine the actual level of feedback he or she prefers to work with (i.e. the predefinition of the gain of resistance in the instrument handle). For this study, the level of feedback was predefined through a face validity test with two laparoscopic experts. Participants performed three series of blinded palpation tasks, involving three different graspers: a conventional grasper and the FROI grasper (Fig. 2), which was used in the activated and deactivated states. In the deactivated state, the FROI enabled force registration without the use of enhanced haptic feedback. Each series involved nine pairs of porcine organ tissues. Through blinded palpation, participants had to compare the tissue consistencies of the two presented tissues and determine which tissue had the most solid consistency. The comparison could involve slices of two different organs (e.g. lung versus liver) or slices of the same organ. Participants were not restricted in the number of palpations of the tissues or the total palpation time. The orders of presenting both the instruments and the tissues were randomized between subjects following a randomized controlled crossover design. A computer-generated randomization was executed with block size 3 and list length 60 for the randomization of instrument order, and block size 9 and list length 540 for the randomization of tissue comparison. A single-blind approach was applied for the palpation of tissues. Blinding for the instruments was not attainable due to the design of instruments and experimental setup.

### Data collection

To record all reaction forces (concentrated load, in Newtons [N]) on the instrument tip of the FROI device (activated and deactivated), the optical signals in the instrument were measured with a Deminsys interrogator. A Spartan-6 field programmable gate array and an Arduino Mega 2560 controller were interposed to enable reading the forces with a Python script and storing the data on a Windows PC. The forces were recorded at a sampling rate of 10 samples/s. Participants determined which tissue they thought had the most solid consistency, and after each palpation, they recorded their assessment on an answer form. The answer form included the options ‘left’, ‘right’, or ‘no difference’. Additionally, participants had to rate their level of certainty on a five-point Likert scale, ranging from very unconfident to very confident.

### Data analysis

All files were analysed by means of a protocol written in MATLAB R2014b (The Mathworks, Inc., Natick, MA, USA). All peak forces were selected, and the average peak force was calculated per grasper for each subject in a series of palpation tasks.

### Statistics

Statistical analyses were carried out with IBM SPSS 22 (SPSS, Inc., Chicago Ill, USA). To determine whether the use of the FROI mechanism had an effect on the applied force during tissue manipulation, we performed a paired samples *t* test. To determine whether the use of the FROI had an impact on tissue recognition and confidence in the answer, a Generalized Estimating Equations (GEE) analysis was performed. For tissue recognition, the outcome was modelled as a function that included the type of grasper, the type of tissue, and the interaction between the grasper and the type of tissue. For confidence, the outcome was modelled as a function that included the type of grasper and answer correctness [data are presented as odds ratios (OR) with 95% confidence interval (CI)]. For both analyses, a *p* value <0.05 was considered statistically significant.

## Results

In total, 7 residents (6 females) and 13 medical students (8 females) participated in this study. Residents had an average of 3.5 years of laparoscopic experience.

### Force application

**Fig. 3:**
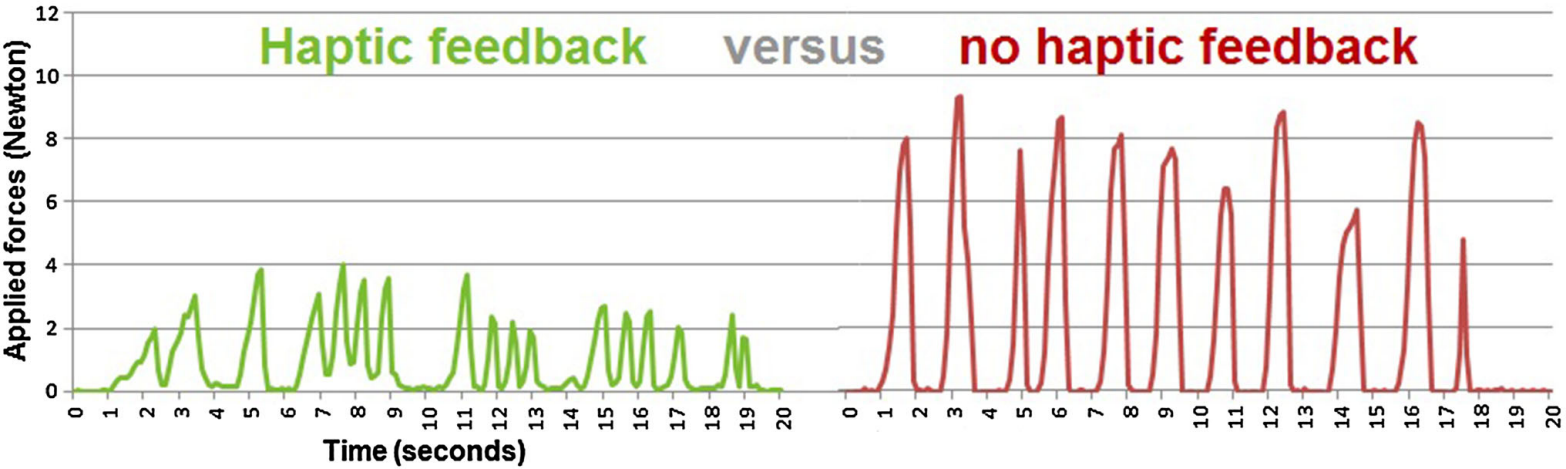
Force application during tissue palpation. These recordings of one participant show forces applied with the FROI with enhanced haptic feedback activated (*left*) and forces applied with enhanced haptic feedback deactivated (*right*)

Due to a technical error in the data-acquisition software, data on force application were incompletely stored in eight cases. To prevent improper data interpretation, we only analysed the force application data collected from twelve participants. There was compliance with the assumption of normality which allowed the use of the Paired Samples *t* Test. On average, the applied force was lowered by a factor of 3.1 (SD 0.4) with the enhanced haptic feedback feature, compared to the conventional situation. The direction of this effect was consistent for all participants, regardless of their experience and the type of palpated tissue. Overall, during palpation, participants applied average forces of 4.6 N (SD 1.5) without haptic feedback, and 1.7 N (SD 0.7) with the addition of haptic feedback. This difference in applied force (2.9 N, 95% CI 2.0–3.8) was significant (*p* < 0.001). In Fig. 3, two graphs depict the typical force application during a palpation task.

### Tissue discrimination

Table 1 shows the percentages of correct assessments in the tissue consistency comparisons for each grasper and for each type of comparison. In cases where the participant palpated two slices of the same tissue, no significant differences were found in the outcomes between the different types of graspers. However, when the participant palpated slices of different tissues, both the activated FROI (*p* = 0.027) and the deactivated FROI (*p* = 0.008) provided significantly enhanced performance compared to the conventional grasper. There was no significant difference for the activated FROI compared to the deactivated FROI (*p* = 0.297).

**Table 1:** Percentage of correctly assessed tissue consistencies for each grasper and each type of comparison

Tissues compared	Conventional	FROI activated	FROI deactivated
Different tissues	52 (42–61)	63 (53–71)	69 (60–78)
Equivalent tissues	47 (35–58)	40 (28–53)	48 (34–63)

Data represent the estimated mean percentage (95% confidence interval). Percentages are based on all 540 cases (20 participants; 9 assessments; 3 instruments)

### Confidence in assessments

**Fig. 4:**
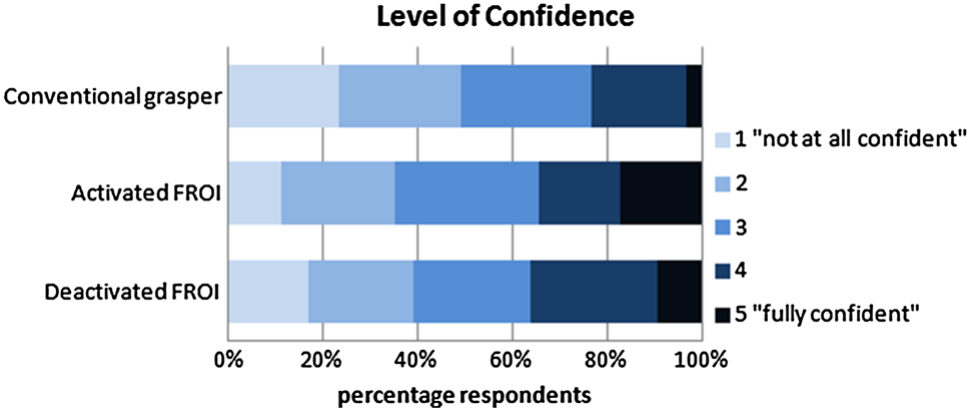
Distribution of the level of self-reported confidence on a 5-point Likert scale for each type of grasper

Figure 4 shows the 5-point Likert scale data for confidence per grasper. The use of the activated FROI was associated with a higher odds ratio for more confidence when compared to both the conventional grasper (OR 1.9, 95% CI 1.4–2.4, *p* < 0.001) and the deactivated FROI (OR 1.4, 95% CI 1.1–1.8, *p* = 0.022). Overall, we found that correct assessments were associated with a higher odds ratio for level of confidence compared to incorrect assessments (OR 2.2, 95% CI 1.7–2.8, *p* < 0.001).

## Discussion

In this study, we examined whether enhanced haptic feedback in laparoscopic graspers could affect hand-tool-tissue interactions. We found that the addition of haptic feedback resulted in an average of 3.1-fold less applied force on the tissue. Furthermore, the use of the FROI resulted in better tissue discrimination and higher confidence in decision-making.

Our finding that haptic feedback in the laparoscopic grasper resulted in significantly less force on the tissue was consistent with findings in previous studies [22, 24, 25]. Because the palpation of tissue in our study was performed in a blinded manner, the effect was not influenced by visual feedback. Lowering the applied force on tissues may have several clinical implications. First, less force is likely to lead to less tissue trauma. A recent study on integrated tactile feedback in robotic surgery on the porcine bowel has shown that this feedback led to a significant decrease in the grasping force and in the overall incidence of tissue damage [26]. A study by Heijnsdijk et al. showed that the mean laparoscopic force applied in bowel handling was 6.8 N, whereas the force required to prevent slippage was 3.0 N [22]. During long laparoscopic procedures, such as hemicolectomy or hysterectomy, a reduction in applied forces is likely to lead to less tissue trauma and, possibly, faster recovery for patients. From the surgeons’ perspective, reduced force application will likely result in a reduction in physical fatigue and, in the long term, a reduction in musculoskeletal disorders due to strenuous surgery. The presence of fatigue or musculoskeletal disorders during surgery has increasingly become recognized as a cause for impaired quality in laparoscopic surgical care [27, 28]. Ideally, both patients and surgeons will benefit from instruments with haptic feedback.

We observed that the use of the FROI, whether activated or deactivated, resulted in better ability to discriminate between palpated tissues. In an earlier study, experienced laparoscopic surgeons stated that the expected advantages of haptic feedback were, among other things, an ability to feel differences in tissue consistencies and in the amount of force applied [17]. The recognition of tissue characteristics will probably be of benefit in surgical procedures such as ovarian cyst removal, malignant disease staging, deep infiltrating endometriosis treatment, and bowel surgery. Furthermore, this feature may facilitate a laparoscopic option for indications that up to now required open abdominal procedures, e.g. surgery that requires lymph node palpation. Haptic feedback in laparoscopic surgery is expected to result in fewer conversions to open surgery. It should also be highlighted that, compared to the conventional grasper, the FROI performed better in tissue recognition, even when the haptic feedback option was switched off. This advantage probably resulted from the low internal friction inside the instrument. However, the FROI was only superior for tissue recognition when there was a difference between the presented tissues.

The third goal of this study was to investigate the effect of enhanced haptic feedback on confidence in decision-making, which is a valuable parameter regarding any human-product interaction. When the tissue discrepancy was correctly determined, we observed that haptic feedback significantly improved the level of self-reported confidence on a 5-point Likert scale. For patient safety, it is of specific interest to determine differences in the level of confidence associated with correct assessments versus the confidence associated with incorrect assessments. Clearly, it is important to avoid great confidence in an erroneous assessment. The present experiment enabled us to link the confidence level to task performance. For all graspers, we found that the level of confidence was significantly higher for correct determinations than for incorrect determinations.

There was little difference between FROI activated and deactivated regarding tissue consistency discrimination and confidence as shown in Table 1 and Fig. 4 respectively. However, from our data it can be concluded that better differentiation already benefits from eliminating the internal losses within the instrument (friction and play). Although not very likely, additional studies will have to reveal if a type II error could have occurred. Furthermore, better or more careful tissue handling solely resulted from the haptic feedback modality activated, as shown in the typical example in Fig. 3.

Although the current generation of laparoscopic surgeons has not received any formal training in laparoscopic instruments with haptic feedback, it is expected that current residents are more acquainted with this type of instrument. Several studies have reported on the introduction and validation of (virtual) laparoscopic training systems with haptic and force feedback [29–32]. In their review on haptic feedback simulations, Pinzon et al. concluded that force feedback was the best method for tissue identification, and that haptic feedback provided the greatest benefit to surgical novices in the early stages of their training [31]. Prasad et al. compared laparoscopic novices and experts and found that novices applied large forces compared to expert surgeons. Furthermore, they found that visual and haptic feedback improved the performance of residents [29]. Therefore, the implementation of haptic feedback in laparoscopic training programmes will most likely benefit skills training, and consequently, laparoscopic performance and patient outcomes. The exception might be laparoscopic suturing, which appeared to be learned more readily in conventional box trainers than in virtual reality systems with haptic feedback [33].

This study had some limitations. First, the number of participants was rather small. Furthermore, due to software issues, not all force patterns could be evaluated. Also, we did not find different results between laparoscopic residents and students; both groups applied the same (high) forces with graspers that lacked haptic feedback. This result was probably due to the fact that both residents and students used the FROI for the first time in this experiment. Lastly, several porcine tissue pairs might not have differed from each other sufficiently to allow definite discrimination.

Several directives for future research can be derived based on the knowledge obtained in this study. Future studies could address the speed of decision-making, which was not tested in this study. Also, future studies should separately address the advantages of haptic and visual feedback, to enable clear distinctions between the added values of these two effects. The technique implemented in the current study was performed with a conventional laparoscopic grasper with the well-known scissor-like hand grip; this grasper enabled a comparison between haptic feedback and no haptic feedback with standard equipment. For future studies, current knowledge on the ergonomics of several hand grips should be taken into account.

In conclusion, we found that the FROI as a haptic feedback laparoscopic grasper enabled surgeons to handle tissues with significantly reduced interaction forces between the instrument and tissue. The observed force reduction is expected to have multiple important clinical implications, including less tissue damage, fewer complications, shorter operation times, and enhanced ergonomics. Future in vivo studies are needed to validate the anticipated clinical benefits.
